# Rethinking well-being in capitalist value-centered societies: football in Japan as contemporary capitalistic ritual

**DOI:** 10.3389/fspor.2026.1717103

**Published:** 2026-04-17

**Authors:** Yosri Razgui

**Affiliations:** Graduate School of Intercultural Studies, Kobe University, Kobe, Japan

**Keywords:** well-being, belief systems, neoliberalism, rituals, football

## Abstract

This paper aims to reflect upon the cultural impact of neoliberal capitalism on the experientiality of contemporary rituals as potential sources of well-being. After modeling a theoretical framework that positions the concepts of rituals, well-being and development within the contemporary context, the paper focuses on the ontology of contemporary capitalistic rituals, a newly proposed category that arguably better situates many modern rituals within the current global context. In conclusion, drawing on an ethnographic case study among Japanese professional football supporters, the paper demonstrates how sports mega-events can be categorized as contemporary capitalistic rituals and play a central role in modeling well-being and happiness in urban settings.

## 1 Introduction

The notions of development and well-being are almost irreparably embedded in a discourse often dominated by economic logics that revolve around the idea of growth: whether it is economic, financial, urban or societal, development is often not treated as such if figures and numbers do not keep rising. From a cultural anthropological standpoint, when asked about how to understand the major causes of the centrality and the hegemony of economic discourses around development, it is imperative to widen the focus of analysis and to identify paradigm shifts of hegemonic values and ideologies that reshape societies both globally and locally. On this note, there is a rich ongoing debate about determining how capitalism, especially in its neoliberal form, has contributed to remodeling the core set of values that structures many contemporary societies. This critique of capitalism as a value provider produced a series of analyses that led to an understanding of it as something that cannot be reduced to a mere economic system. Mark Fisher's iconic *Capitalist Realism* (2009) highlights its pervasiveness and embeddedness in our thoughts and actions, suggesting that capitalism has a primary role in defining visions of the future and perceptions of the world. In a similar vein, David Harvey and Nancy Fraser recognize capitalism as functioning as a value-providing ideology (1) and an institutionalized social order (2).

The human being, as a symbolic animal, is distinguished from other living beings by its ability, or even necessity, to symbolize, to establish morals and values and to draw meaning from the intricate fabric of symbols and ideas that structure entire ideologies and belief systems. Hence, situating capitalism as the major value provider for a large part of contemporary societies implies a series of consequences: 1) assuming that capitalistic worldviews are now deeply embedded and institutionalized in our social order, capitalistic values are supposedly shared and spread through institutions such as schools, families and governmental bodies, shaping perceptions of life from childhood; 2) core capitalistic values such as careerism*,* individualism, competition and capital accumulation affect humans' process of symbolization, and thus their ability to find meaning and purpose in life, as well as the choices they make in attempting to fulfill them; 3) notions that heavily rely on processes of symbolization, such as taste, cultural capital (3, 4), happiness and well-being are directly exposed to the capitalistic worldview and to its symbolic hegemony, which assumes the role of filtering most symbolic and cultural activities. This results in the need to readapt the analytical schemes used so far to interpret human behavior at both individual and collective levels.

Through prior research (5), this line of reasoning led me to the conclusion that if capitalism is an ideology, an institutionalized social order, and a value provider, then one could argue that capitalism could be best understood as a belief system, an existential mechanism that structures people's symbolic activities by providing them with new symbolic ethos and horizons while being sustained by human symbolic activities such as myths, legends, heroes, and rituals. Belief systems, such as religions, ideologies (political, economic etc.), and philosophies, have a close relationship with the pursuit of happiness and well-being. Throughout history, humans proved themselves to be great thinkers, philosophers, and story tellers, symbolic abilities that helped generation after generation to find solutions to complex existential problems. Cultural anthropology's comparativism has shed light on how religions function very well at finding explanations for unsolvable questions, helping believers make sense of existential disorientation, suffering, trauma and despair. Thus, we can argue that belief systems not only can serve as a response to an urge to find ease by getting answers to existentially problematic dilemmas and questions, but they actively provide humans the possibility to adhere to structural symbolic mechanisms, life patterns and sets of myths, legends and rituals that could define and provide solutions for the pursuit of happiness and well-being. Neoliberal capitalism can be seen as a belief system that fulfills the same functions. In societies based on a capitalist model, individuals’ life trajectories depend deeply on the morals and values established and promoted by neoliberal capitalism. As a result, both collective and individuals’ expectations, desires, and dreams get profoundly shaped by it.

Rituals occupy a central role within belief systems, including neoliberal capitalism. In previous research (5), I introduced the category of *contemporary capitalistic rituals* to describe ritual practices embedded in, and shaped by, capitalist logic. Building on my previous findings, this paper aims to investigate the central role of capitalist belief in linking symbolization processes to perceptions of happiness and well-being by drawing on an ethnographic study of football^1^ supporters of a professional club in Japan. After outlining the methodology used for the study, the work presents a theoretical framework that encompasses the reflexive relationship between rituals, capitalism and development (intended here as the pursuit of well-being). The paper will then provide a brief overview of what is meant by *contemporary capitalistic ritual*, analyzing its entanglement with the contemporary perception of well-being. Lastly, the focus will shift to a case study of Japanese football supporters, with qualitative data used to support the claim that neoliberal capitalism and *contemporary capitalistic rituals* play a central role in the pursuit of well-being and societal development.

## 2 Methodology

Given the lack of qualitative research on sports fandom, various scholars have been trying to fill this gap through fieldwork and ethnographic research over the past few decades (6, 7). These studies demonstrate the efficacy of interpretive and immersive methods in shedding light on the cultural dynamics that structure sport-related phenomena and their significance in wider societal matters. This work, which follows the same trajectory, adopts a qualitative approach that moves along two main axes: ethnographic fieldwork, grounded in observant participation, and an interpretative, thick-description framework (8, 54).

In line with contemporary interpretivist approaches, the aim is not only to document practices but to understand the *meaning-making processes* through which supporters negotiate identity, affect, and social belonging in their everyday engagements.

The qualitative data used here were collected over an extended but fragmented period of fieldwork conducted among the supporters of Vissel Kobe, a professional football club based in Kobe (Japan). The fieldwork—originally carried out for my doctoral dissertation—resulted in 42 months of observant participation at football venues; hours of informal interlocutions with supporters; semi-structured interviews; and lengthy fieldnotes. The informal interlocutions represent the core body of the collected data, as many of the most significant conversations took place during downtime ahead of kick-off, in travel to football venues, and during convivial, unstructured moments. Alongside fieldnotes, the corpus also features a wide collection of photos and videos documenting supporting activities.

The first stage of fieldwork took place as part of my master's research, from March to August 2017, allowing me to get acquainted with the organized group leading Vissel Kobe's supporting activities, known as *11Stones*. I was kindly welcomed into the group after only a few weeks, becoming a semi-active member and taking part in most of their major activities. At the time, the group included around thirty active members who followed every home and away game, as well as a broader circle of occasional participants. During this phase, I built a solid network with most of the active members—connections that enabled me to return to the field two years later.

The fieldwork resumed in February 2020, after my return to Kobe as a doctoral student, allowing three additional years of continuous observant participation. The data-collection modalities remained consistent, with occasional semi-structured interviews when deemed necessary. The longitudinal nature of the fieldwork proved methodologically valuable: on the one hand, it enabled me to observe features that shifted over time (such as internal structures and long-term projects); on the other hand, it exposed the challenges of “ethnographic stagnancy,” including the progressive decrease of novelty and the changing ways in which interlocutors related to my presence.

Although the main venue of the fieldwork was Noevir Stadium (also known as Kobe Wing Stadium), participation in an organized supporters’ group necessarily entailed a multi-sited approach. Supporting a football club requires constant travel to stadiums across Japan (away games), and the use of a variety of spaces for organizational purposes, consumption practices, and ritualized conviviality. Although focused on one team, the fieldwork allowed me to observe, albeit from some distance, supporters of different clubs and geographical contexts. This multiplicity of sites and encounters reinforced the interpretive dimension of the research by situating practices within broader networks of mobility, affect, and ritual engagement.

The qualitative corpus used in this article has already been partially employed in a previous work (5) focused on the ritual dynamics of Japanese football. The present article departs from that earlier work by introducing a secondary analytical layer: in revisiting my fieldnotes and audio-visual materials through the lens of contemporary debates on neoliberal capitalism, development, and well-being, I adopt an interpretive position aligned with anthropological approaches that recognize the *multi-temporality* of ethnographic data (9, 10). Distance from the field, as well as the evolution of anthropological debates on neoliberal affect, consumerist well-being, and development ideology (11, 12), has enabled a reflexive re-engagement with the corpus. Rather than imposing an external framework, the analytical turn emerged from recurrent themes within the material itself: conversations about work fatigue, the emotional pressures of urban life, and the ritualized search for well-being and communal relief around football events.

For this reason, the same ethnographic corpus is employed here not to repeat descriptive accounts, but to develop a theoretical framework that interprets contemporary supporter practices as cultural sites where neoliberal logics are both reproduced and negotiated. This methodological approach triangulates long-term ritual ethnography with recent anthropological notions about well-being and development, therefore tracing the conjunctures between neoliberal culture, development discourse, well-being, and sport-related contemporary rituals.

## 3 Development and the neoliberal way to well-being

Before entering the core of the discussion proposed in this paper, it is necessary to clarify how the notions of development and well-being will be tackled in this reasoning. For a flexible and tentatively unbiased definition, the one used by Marc Pilkington (2015) for his essay on well-being, happiness and the structural crisis of neoliberalism aligns well with the purpose of this paper:

“[Well-being is] a dynamic state, in which the individual is able to develop their potential, work productively and creatively, build strong and positive relationships with others, and contribute to their community. It is enhanced when an individual is able to fulfil their personal and social goals and achieve a sense of purpose in society (Government Office for Science, 2008).” (13)

When it comes to development, anthropological debates have helped to identify the positionality challenges that one might encounter when labeling this notion from an academic point of view. While tackling the relationship between anthropology, sport and international development, Brownell (14) highlights the importance of reframing the notion of development starting from the question “Who defines what development is?”. As anthropologists pointed out, the notion of development is nowadays strictly entangled with an occidental-centric worldview that sees the “developed” Global North as an entity that should help “develop” the Global South (14, 15), suggesting that development is something that can be attained exclusively through economic growth. Considering these current tendencies of global economies and politics, one could assert that development is widely seen, both popularly and academically speaking, as something intrinsically related to neoliberal capitalism.

What is interesting is that this notion of development is nowadays frequently presented as being synonymous with well-being, an equivalence that, upon closer examination, reveals itself to be both ideologically constructed and empirically unsustainable. The rhetoric of development, particularly as it has been articulated in global governance, international aid, and policy frameworks, tends to mask its underlying prescriptive nature: the imperative to adopt capitalist modes of economic organization and to integrate into financial systems structured and controlled by leading capitalist states. In this sense, development often functions less as a neutral process of societal improvement and more as an invitation, or, in many cases, a coercion, toward economic and political subordination.

When reframed in terms of well-being, the capitalist project exposes a profound internal contradiction. On the one hand, capitalism constructs and regulates the symbolic lexicon through which happiness is imagined, narrativizing the acquisition of wealth and social mobility as the ultimate pathway to personal fulfillment. This is epitomized in the romanticized “self-made man” (16, 17), whose disciplined labor and entrepreneurial spirit are celebrated as both moral virtues and vehicles for upward mobility, thereby reinforcing the equation that frames economic growth as development. On the other hand, capitalism's structural operations inevitably produce widespread material deprivation (2), systematically concentrating wealth in the hands of a few, while leaving vast populations in conditions of precarity or outright poverty. For these individuals, the capitalist definition of happiness—as something contingent on wealth—becomes both a normative ideal and an unattainable horizon, producing a condition of chronic dissatisfaction and, by extension, the negation of well-being.

Participation in capitalism, whether voluntary or coerced, tends to involve the internalization of its symbolic grammar. Over time, this symbolic system comes to mediate not only the means but also the ends of well-being, embedding happiness within the capitalist structure as both an aspirational goal and a mechanism that sustains the system itself. Within this framework, well-being is often pursued along two primary trajectories: a systemic one, wherein fulfillment is sought through occupational achievement, income generation, and the accumulation of social capital through economic status; and a less systemic one, wherein work is rejected as the central source of well-being and leisure can become a ritualized space for its pursuit—through practices such as fitness, beauty care, tourism, or participation in cultural and entertainment events. However, despite being anti-systemic, the latter trajectory seems to be quite prevalent in capitalist societies, where dissatisfied workers seem to outnumber those who experience emotional fulfillment through their jobs. This highlights a great contradiction of the neoliberal mission: on the one hand it spurs individuals to follow their individual desires and find the “dream job”, but on the other hand it does not provide a societal structure that allows it. Drawing from David Graeber's work, the result is that a wide portion of individuals find themselves dealing with unfulfilling jobs (18), making “free time” a central part of human existence, a ritualized space where alternative existential ethos can be negotiated. Yet, even outside the systemic capitalistic way of happiness, the pursuit of well-being in contemporary times remains tethered to the circuits of capital, as the goods, services, and infrastructures enabling leisure practices are often products of, and contributors to, capitalist accumulation. In this way, the very grammar through which well-being is imagined ensures that it remains inseparable from the economic and cultural system that both defines and constrains it.

## 4 Rituals, capitalism and well-being: a theoretical framework

Despite the fact that most societies have supposedly shifted toward more secular and religionless structures, rituals are still widely researched and analyzed as a central symbolic mechanism in people's lives (19). In fact, numerous scholars are still conducting research on how rituals are interrelated with life-cycle transitions, emotional regulation and processing, social cohesion and community building, identity formation, norm and social order reinforcement, and many other aspects that characterize human experience (20–22). This shows how rituals, rather than belonging to the so-called “traditional” sphere, contribute to the dismantling of the essentializing traditional/secular dichotomy, establishing itself as a trans-historical and intersocietal existential behavior that defies culturally and historically constructed paradigms. However, despite the ongoing interest in rituality, the excessive xenophilia that sometimes pervades anthropological inquiries have caused contemporary issues (or for a more provocative term, “western” issues) to be disregarded or even labeled as not worthy of inquiry. A clear example is how the ludic sphere (e.g., leisure, games and sports) had been ignored for decades before becoming a serious object of research during the second half of the twentieth century. One of the first ethnographic studies fully dedicated to football culture as a *total social fact* (23) dates back only to 1995, when the French anthropologist Christian Bromberger published his ethnographic work on a selection of European supporters. Unfortunately, even though anthropological research about contemporary sports have become significantly more popular since then, there is a clear lack of studies based on ethnographic support.

Another aspect of contemporary societies that lacks academic inquiry is the entanglement between capitalist culture and ritual life. Although many scholars have identified consumption as the new central behavior around which a large part of contemporary ritual activity revolves (24, 25), they tend to treat it as the only aspect of neoliberal capitalism that is ritualized, thereby implying an overlap between capitalism and consumerism. As argued by Varul (26), consumerism, more than a direct consequence of the capitalistic logic in its Marxian sense, is an ideological tool that specifically made neoliberal capitalism the most pervasive and embedded form of capitalism ever. This focus on consumption led to a tendency to overemphasize it as the center of symbolic interactions between individuals, consequently raising the risks of overlooking other fundamental aspects of the capitalist structure that dominate more pervasively every aspect of human life (time perception, value formation etc.). It is for these reasons that, building on the works of authors like Rejman and Czubocha (27) and Vincenzo (2016) who tackled capitalism and consumerism as a culture-based semiotic machine, I argue that what is currently lacking in this discourse is an up-to-date theoretical framework that explains how the neoliberal capitalist structure functions as a symbolic frame where individuals draw new meanings and values for their cultural activities. Given this gap, I argue that capitalism should be seen as a belief system with its own set of myths, legends, values and, most importantly for this dissertation, rituals. All these aspects strongly contribute to the symbolization of everyday actions and to both individual and communal engagement, thereby playing a central role in shaping perceptions of happiness and well-being.

### 4.1 Rituals as sources of well-being

With regard to rituals and well-being, ritual theory has already addressed the role that some ritual aspects, such as *collective effervescence* (28) and *community building*, have with the building of sense of happiness and well-being:

“Ritual practice generates emotional effervescence, a cross-culturally known experience of transcendental feeling that gives rise to religious life as well as to the establishment of ideals. Numerous contributions to the anthropology of ritual have acknowledged the significance of positive emotions like joy and the pleasure in sharing common experiences with like-minded people for the forging of communitas [e.g., (29)].” (30).

But how do these rituals function in a capitalist society? What aspects of society are more exposed to rituality? How do people benefit from rituals in capitalist contexts? To address these questions, it is important to first provide a clear overview of what being a capitalist society implies. Instead of conducting a systematic analysis, this discussion will focus on elements that can contribute to a better understanding of the implication of rituals. First, contemporary capitalist societies are based on an economic system in which the means of production are generally privately owned, marking a clear distinction between a class of capitalists who monopolize the means of production and a class of workers who do not have direct access to them. As Karl Marx highlighted, one of the central features of capitalistic working culture is that the worker is not paid according to the value of the products generated through their labor, but rather on a time basis. These two structural aspects of capitalism have contributed to establishing another major structural feature: the separation between working time and “free time” (or “leisure time”), a separation that has intensified since the Industrial Revolution, when wage labor replaced many forms of subsistence work (31). Capitalist societies are therefore generally organized around this time-perception framework, one that, from the point of view of the worker, is divided into multiple complementary cycles: weekly cycles (weekdays vs. weekends), yearly cycles (working days vs. holidays), and market-based cycles (economic growth vs. recession). Although working culture has recently shown new traits that challenge the normative capitalistic division of time (flexible work, gig economy, remote work etc.) it can still be asserted that the normative capitalistic perception of time represents the vast majority of such societies.

This cyclical division between “working time” and “free time” has largely replaced the temporal systems that were central to pre-industrial societies. With this paradigm shift, older rituals slowly adapted to the new symbolic grammars established by the hegemonic capitalist belief system (a process generally referred to as *secularization*) while, at the same time, new rituals emerged from capitalist moral and value schemes. To give respective examples: the current way religious celebrations, such as Christmas in Christianity or Eid al-Fitr (marking the end of Ramadan) in Islam are celebrated show how even those practices that were considered highly spiritual and religious have been absorbing consumerist symbolic elements (32, 50). On the other hand, among the newly generated rituals, some of the most iconic and widely studied are those related to consumerist culture, a key pillar of neoliberal capitalist societies (24, 27). Regardless of the novelty of the ritual, it is important to point out that a vast majority of ritual practices in contemporary capitalist societies take place, for obvious reasons, during the “free time” slots, making “free time” a highly symbolic space where various kinds of ritual activities occur. This can be seen as a proof of the fact that the ritual life, especially referring to those rituals symbolically linked with leisure and entertainment, had to readapt to the capitalistic perception of time, to the capitalistic ideologies and to the current values that structure this hegemonic capitalistic belief system.

**Figure 1:**
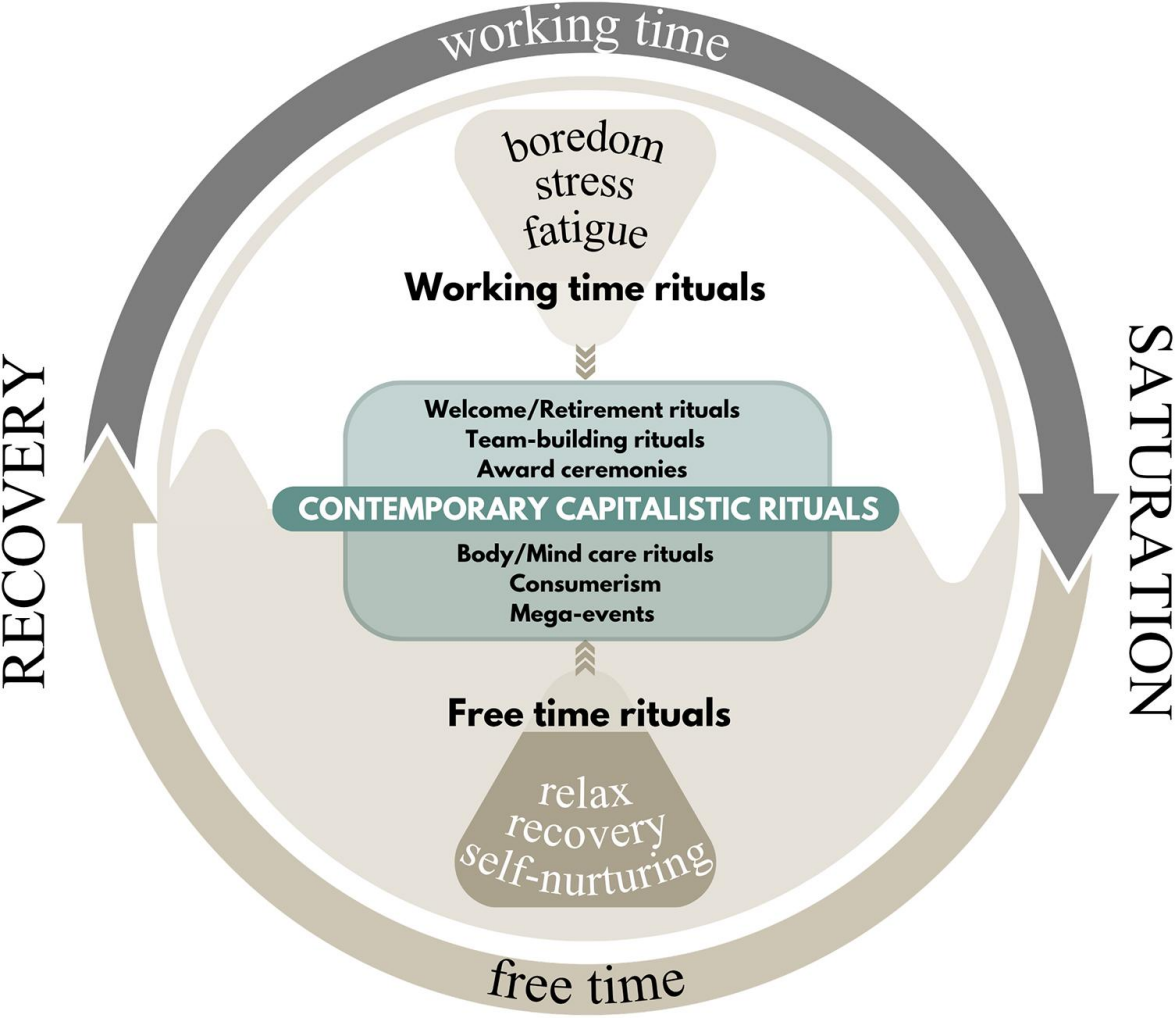
The structural time cycle in societies where neoliberal capitalist values are hegemonical.

To facilitate the understanding of the rituals that fall within this framework, I propose a new category, which I define as *contemporary capitalistic rituals*. This category groups those rituals that function to maintain or reinforce the capitalist social structure, which rests heavily on the dichotomous work/leisure-time framework. *Contemporary capitalistic rituals* thus encompass, in principle, all ritual practices that play a role in symbolically structuring both temporalities of capitalism (Figure 1). At one level, there are those rituals that accompany the individual along the path of embedding capitalist values, ensuring that they become a fully functional adult within the system, from schooling to incorporation into corporate culture. At another level, the rituals of free time provide the necessary window for psychophysical recovery, allowing individuals to remain functional within the capitalist cycle. These can be understood as a subcategory of “*free time rituals”* (5), and they are generally symbolically correlated with notions of well-being, happiness, and relaxation. Such rituals are diverse in nature and can manifest in multiple forms: from excursions into nature (rituals of purification), to re-aggregation with loved ones (rituals of re-aggregation), and even the purchase of material goods. The centrality of “free time” of the capitalist model and the collocation of most of well-being-related rituals in this span of time require academic inquiry to delve deeper into the ritual role of the “free time” as something that opposes “work time”. Consequently, when tackling the notion of development of well-being it becomes crucial to understand the characteristics and the cultural mechanisms that govern within this structural dichotomy.

To support the proposed argument, the next section introduces a case study from Japan. It begins with a brief historical overview that highlights the strong connection between consumerism and the sporting event, a feature that has characterized Japanese professional football since its inception. The Japanese case is particularly well suited to examining the ritual entanglement between football and neoliberal capitalism, because it is one of the few contexts in which the emergence of professional football resulted from a deliberately orchestrated, step-by-step financial strategy rather than from a slow, diachronically layered process of historical stratification. The paper then presents ethnographic evidence to show how *contemporary capitalistic rituals* can play a significant role in the pursuit of well-being and happiness within a neoliberal context.

## 5 Historical context: J.League as a “neoliberal signifier”

With the exception of baseball, whose widespread and almost immediate popularity facilitated its professionalization in 1936, most sports—particularly football, basketball, and volleyball— had for decades remained bound to the school–work system and amateur contexts through *bukatsu*^2^. The decision to permanently transform Japanese football dates back to 1989, when several members of the JFA (Japanese Football Association) commission (Naganuma Ken, Murata Tadao, Okano Shunichirō, and Kawabuchi Saburō) launched the J.League (Japan Professional Football League) project. The initiative entailed an investment of 20 billion dollars and a national development plan for Japanese football (33). Beyond professionalization, the JFA also set its sights on bidding to host the 2002 FIFA World Cup. This meant that, in addition to the institutionalization of football, substantial infrastructural investments would be required to build new facilities and stadiums in order to meet FIFA's standards. At this stage, football was also framed as a tool for the “development” of peripheral areas through infrastructural intervention. By introducing a new form of entertainment and generating collective symbols tied to these regions, football was expected to exert a substantial influence on decentralized areas (53). Given the ambitious and costly nature of the project, investors would not have accepted the risk without the marketing strategies that promised economic returns from the establishment of the J.League. This constitutes a crucial point for understanding the nature of Japanese football: unlike in many other contexts, where the professionalization of football can be described as the outcome of a gradual and reflective process shaped by different historical, cultural, and economic phases, Japanese professional football emerged as the result of meticulous financial planning. This planning was aimed both at sparking a kind of cultural revolution in the practice—enabling Japan to integrate into the global football arena—and at creating a financial apparatus capable of capitalizing on a new commercial product to be offered to the public (34, 35).

The J.League was inaugurated at Tokyo National Stadium (*Tōkyō Kokuritsu Kyōgijō*) on 15 March 1993 with a match between Yokohama Marinos and Verdy Kawasaki. The widespread curiosity surrounding this new mega-event confirmed the effectiveness of a strategy aimed at launching and legitimizing a new product within a sports consumption market long dominated by baseball, a discipline with a far more established professional history. Even before the opening match, the J.League had already become a brand conceived to progressively integrate into the everyday lives of future fans/consumers. The J.League label was omnipresent: J.League chips, J.League chocolate, and many other products (35). Major corporations quickly perceived its vast economic potential, offering their support as sponsors of the project. Symbolic in this regard was the decision to name the competition the Suntory Series for the first half of the season and the Nicos Series for the second, after the respective beverage company Suntory and credit card company Nicos (35). Naturally, the marketing campaign continued at full speed in the years following the debut of the new professional league. For example, in its first year, the Japanese electronics giant Sony developed approximately 1,700 J.League-branded products. The sportswear brand Mizuno sold more than one million flags, along with hats, scarves, and numerous other fan-related items (35). Jonathan Watts, an English journalist who closely followed the birth of the J.League, employed the concept of *shinhatsubai* to describe the novelty of the football phenomenon, noting that the marketing techniques used to promote the league resembled standard corporate procedures for launching a new product: “These new updated and re-packaged goods are marketed under the label *shinhatsubai*, a term which corresponds in English to “new release”. […] The expression conveys a sense of a new movement of goods to the marketplace.” [(36), p.183]. Beyond merchandising and large-scale promotion through a dense network of sponsorships, the professionalization of the J.League also relied in part on a process of “exoticization,” aimed at rendering football a unique and innovative product—particularly with the intention of creating a market alternative to the segment already dominated by baseball.

Marketing strategies were not limited to the mass production of merchandise and sponsorship products. They even extended to design policies concerning the purely competitive dimension and the technical-tactical development of football in Japan. For instance, the recruitment of internationally renowned coaches and players was intended not only to enhance the commercial appeal of the J.League but also to rely on these professionals for the overall improvement of the national footballing standard. However, as Moffett observed, it was peculiar that the majority of players brought from Europe or South America were forwards: for the novice fan, not yet able to appreciate the complexities of tactical play, the goal-scoring moment was far more engaging than understanding intricate tactical maneuvers, which at first glance appeared dull (35). Although the “exoticizing” function of these foreign faces was significant, other factors must also be considered in the broader relational context between Japan and the rest of the world. The Meiji period (1868–1912) was characterized by intense intercultural exchanges across multiple domains. Despite the heterogeneity of knowledge circulating to and from Japan, a common thread of the post-isolationist period was the tendency to position oneself a step below “Western” realities, a tendency encapsulated in the expression *wakon yōsai*, translatable as “Japanese spirit, Western knowledge” (37). The recognition of what was perceived as a “delay” in technological, economic, social, and political development ushered in a phase of intensive study of “Western” expertise, with the aim of bridging the gap and becoming competitive on the international stage. This aspect also shaped the history of Japanese football, particularly from the Tokyo Olympics of 1964 onward—the first major encounter between Japanese and international football. Kawabuchi Saburō, then a striker for the Olympic team, would later become the first chairman of the J.League, and it was precisely from that experience that his ardent desire to improve Japanese football through professionalization was born. Ahead of Japan's first Olympic Games, the team was placed under the guidance of a German coach, Dettmar Cramer, whose task was to provide professional football lessons to amateur players affiliated with the *bukatsu* (clubs) of the corporations participating in the JFL championship (35). A training camp was even organized in Germany, where the young Kawabuchi was struck by the level of organization required to ensure a club's competitiveness. The seed of football professionalization was planted in that moment, only to blossom in the 1990s with the revolution led by Kawabuchi himself. Returning to the early years of the J.League, beyond the already discussed financial-economic dimension, the idea of recruiting foreign players in the twilight of their careers stemmed precisely from the need for highly experienced professionals who could “teach by playing.” These strategies enabled the J.League to achieve immense success in its early years. Although public interest later experienced highs and lows, on the whole—and thanks to international events and the achievements of the national team—Japanese football appeared to have reached a sufficiently high level of popularity to ensure its long-term success.

## 6 J.League and well-being

As evidenced by the earliest sociological studies of football—often centered on issues of civil disorder (hooliganism) (38–40), discrimination (racism, misogyny) (41, 42), and the infiltration of organized crime groups (43, 44)—European and South American football has frequently been associated, academically but above all popularly, with an inherently anti-structural world of anti-social effervescence. From a semantic standpoint, this type of association has long positioned football culture within the sphere of *ill-being* rather than within those of happiness and well-being. However, an examination of the narratives employed by the J.League and the JFA in launching the new cultural-financial product of the J.League makes evident the intention to “symbolically gentrify” a culture that for many decades had been linked to violence. This intention is clearly expressed in the official statements of the J.League and the JFA. On the J.League's website, among the project's missions one finds the following statement:

“To foster the development of Japan's sporting culture, to assist in the healthy mental and physical growth of Japanese people.” (J.League Official Website)^3^

In the same vein, among the ideals and objectives of the project are listed:

“Through football, we realise the full benefits that sports can bring to our lives—the soundness of our bodies, the expansion of our minds, and the enrichment of our societies.”

“By bringing the football experience closer, we bring sport itself closer to all; from this affinity, we will create an environment rich in enjoyment and happiness.” (J.League Official Website)

The key phrases *healthy mental growth, healthy physical growth, soundness of our bodies, expansion of our minds, enjoyment and happiness* highlight the explicit intention to semantically link professional football—still a novel phenomenon at the time—to the concepts of happiness, well-being, and mental health, namely the core elements that popularly define the notion of well-being. In other sections of the manifesto, the intention is also expressed to create a safe environment suitable for the enjoyment of the product by all people, without discrimination on the basis of gender or age, with a particular emphasis on families. The message appears directed at Japanese society as a whole, encompassing both those who experience football through direct practice (players and coaches) and those who experience it as spectators (fans, viewers, or consumers). On the one hand, the connection between direct participation in sport and psycho-physical well-being is relatively straightforward (care of the body, healthy diet, collective participation, etc.); on the other, the connection between the “passive” consumption of sport as a spectator/consumer is far less direct and requires a more sustained analytical effort, which I propose in the following paragraphs.

Although the terms *well-being* or *happiness* are not explicitly used, the analytical efforts of earlier anthropologists of sport (45, 55) produced compelling hypotheses that explained the relationship between being a spectator of a mega-event and attaining a certain state of ecstasy and happiness. However, as discussed in the previous sections, such theories were formulated with reference to societies that had not yet reached the degree of neoliberal cultural pervasiveness experienced in the contemporary world (46). Over the last decades, the ideological hegemony of the neoliberal belief system has been reshaping the symbolic sphere of large segments of global society, restructuring the symbolic grammar of many forms of human cultural activity (1). While in certain countries (most notably in Europe) the discussion has often centered on the *commodification of football*, pointing to a symbolic transition from one paradigm to another (47), the Japanese case illustrates how professional football was planned as a product that had to be structured and sustained by this new neoliberal paradigm. This is why the J.League can be seen as an ideal case to be analyzed as a case-study of re-symbolization of both a global culture such as football and of the ways through which one seeks to achieve a state of well-being and happiness. It is precisely this opportunity for re-symbolization that allowed the JFA and the J.League to operate on a semiotic-communicative level and to attempt to redesign the modes of experiencing a football match.

Therefore, one consequence of this cultural paradigm transition concerns the very modalities through which well-being is achieved via symbolic-ritual activities. If in the football matches analyzed by Bromberger (45), euphoria and happiness were seen as the product of collective effervescence, in contemporary football matches the pathways toward such euphoric states appear to have been re-symbolized according to the semiotic grammar of neoliberalism. It is no coincidence that the financial ideals underpinning the launch of the J.League seem to allude, albeit indirectly, to a correlation between a consumerist disposition toward the J.League and the attainment of psycho-physical well-being. This correlation is only possible within the binary conception of time introduced by capitalism. The time of labor, which is simultaneously the time of wage-earning, becomes the necessary precondition for the salaried worker's access to the *contemporary capitalistic ritual*. Not coincidentally, fieldwork among Vissel Kobe supporters revealed that the majority of adult interlocutors who regularly attended matches were salaried workers, that is, individuals who conceive of time cyclically, alternating the weekly days of labor with the football weekend. Exceptions include those individuals “exempt” from wage labor either structurally (women on maternity leave, children, and retirees) or liminally (students, freelance or part-time workers), but who nonetheless rely on sources of sustenance that are either structural (family support, pensions) or less structural (independent income).

In a global society where consumerism has been structurally embedded since the advent of modernity (48), the neoliberal dichotomy opposing work time and free time appears to generate, among individuals particularly exposed to the capitalist structure, a form of identity duality that pits the *working-self* against the *free-time-self*. Anthropology has already discussed how the focus on the self may be interpreted as a consequence of the value hegemony of neoliberalism. Values such as careerism and consumerism (49) manifest themselves pervasively across various levels of human existence. The *free-time-self*, for instance, demonstrates a strong correlation with a persistent consumerist disposition, attributable to multiple factors of a structural nature (I consume because I must), social nature (I consume to fulfill a certain role in society), and identity-related nature (I consume because I am). In this way, the act of consumption, which in neoliberal terms is translated into the act of moving freely within the free market through the deployment of one's personal capital, becomes a fundamental act of the human existential ethos. The definition of social status or one's perceived identity can thus be directly correlated with a sense of well-being, happiness, and relief.

On the other hand, the *working-self* is often associated with feelings of stress, fatigue, and boredom, assuming a complementary relationship with the *free-time-self*. In this way, the capitalist temporal cycle enables the worker to recover energy and well-being through free time, transforming the latter into a ritualized time in which psycho-physical energies are regenerated through various forms of cultural activity (including sports mega-events), thereby theoretically ensuring the productivity of the *working-self*. Although seemingly complementary and distinct, the two temporal modes/identities are equally subject to the symbolic influence of neoliberal capitalism, which shapes not only a wide range of actions and rituals belonging to the sphere of leisure but also those associated with the sphere of labor. For this reason, the values of the neoliberal capitalist belief system can be identified both in the structural domains of society (school and work) and in those realms lying complementarily around or outside the structural sphere (after-school activities, leisure, retirement, and rituals).

The symbolism underpinning this dichotomy appears to extend beyond the temporal sphere, ultimately shaping the spatial dimension as well. For instance, just as with the domain of labor, the modern conception of the city (generally understood as the urban space of the *working-self*) is frequently correlated with the sphere of *ill-being*. The city, the principal stage of the industrial revolution, continues to be popularly associated with negative elements that impinge upon the well-being of both individuals and communities. Within this context, the stadium acquires a liminal role, providing the ritual space necessary for the organization of a mega-event, which in turn functions as a spatial-temporal setting aimed at restoring the well-being of the *working-self* and sustaining the operability of the capitalist cycle.

Of course, mere attendance at a football event, or simply being present within a stadium, is not in itself sufficient to guarantee an experience that produces an increase or restoration of well-being. Within these ritual frameworks, supporters engage in a set of ritualized behaviors that enable them to experience the event positively (or, as will be discussed, “symbolically” negatively). The following sections will outline the two principal modalities through which supporters participate in the football match: the structural way and the anti-structural one.

## 7 Nurturing the self: individual-oriented supporters in the neoliberal scene

This section examines the mode of support (cheering) that I will call *structural*, drawing on the anthropological concepts of structure and anti-structure developed by the British anthropologist Victor Turner (29). The use of the category of *structure* to define this form of fandom stems from the fact that supporters’ symbolic actions within the ritual frame of the football match—understood here as a *contemporary capitalistic ritual*—appear to mirror the symbolism and values articulated by the symbolic grammar of the neoliberal belief system. In this context, the supporter operates symbolically within an individualistic dimension in which market dynamics, economic transactions, and the consumption of material goods play a fundamental role in the very ontology of the supporter. Personal experience, individual will, personal desires, and ultimately one's own well-being become the central focus of the event. Consequently, individual supporters (or small family units^4^), in the absence of a collective ethos regulating behavior, are compelled to ritualize their supporter ethos through individualized symbolic acts. It is in this sense that the purchase of material goods (gadgets, merchandise, etc.) symbolically linked to the club assumes a central ritual significance within the supporter's imaginary.

The elements that compose this consumerist imaginary are numerous and diverse. As discussed in previous sections, the very birth of the J.League implicitly suggested that the ideal mode of experiencing the league was through consumption. This narrative has since been perpetuated over the years by individual clubs, whose management of marketing strategies and merchandising practices has always played a fundamental role not only in their financial stability but in their very ontology. In fact, a club is first and foremost a business that depends on closing its annual accounts in balance. Accordingly, supporters are continuously exposed to consumerist stimuli, both digitally (through official websites, applications, and email newsletters) and on-site at the stadium. Among the first pieces of information fans receive by email prior to a match (basic game details aside) are updates about food stands, new merchandise, and initiatives promoted by corporate sponsors. A similar form of influence unfolds at the stadium itself, where the first things to greet spectators are the various stands neatly arranged around the arena. The pre-match atmosphere is festive, offering supporters a wide range of options for how to spend their time and their money before kick-off—whether sampling local food, browsing the club store for new products, or queuing for the “free” gifts reserved for season-ticket holders or fan club members.^5^

Typically, regular stadium-goers arrive already dressed according to the supporters’ dress codes: a scarf and official shirt for those who are minimalists, while others add further gadgets such as pins, hats, or keychains to display a greater number of club-related items. The frequency of purchasing the official jersey—whose price starts at a minimum of 20,000 yen per unit—depends on the supporter's economic situation. Nonetheless, it is easy to observe that a large share of fans regularly buys the new jersey with each design change (usually once per year, except on special occasions such as anniversaries). At the turnstiles, supporters are greeted by a team of staff distributing flyers or promotional sets summarizing the various initiatives organized by the club's sponsors for that day. Once seated, exposure to advertising continues through the panels surrounding the stadium and the giant screens projecting commercials until shortly before kick-off. In this pre-match phase, the structural supporter assumes a dual role: on the one hand, they are a consumer of the event, as ticket-payer and purchaser of material goods associated with the club; on the other hand, they are themselves *consumed* by the corporate sponsors, who pay the clubs in exchange for the supporters’ forced exposure to their advertising.

The atmosphere changes drastically with the entrance of the players onto the field for their warm-up. Music (typically rock) serves as a symbolic separator between the advertising/sponsorship phase and the sporting event itself. It is precisely with the arrival of the players, the primary targets of supporters’ activities, that fans begin to symbolically perform their support and attachment to the players or to the team as a whole. The type of ritual performance varies according to the areas of the stadium. To simplify to the extreme: *structural support* characterizes the majority of the stadium, with the exception of the area called *gōru ura* (seats behind the goals), generally reserved for organized supporter groups and hardcore fans (to be presented in the next section as *anti-structural support*).

**Figure 2:**
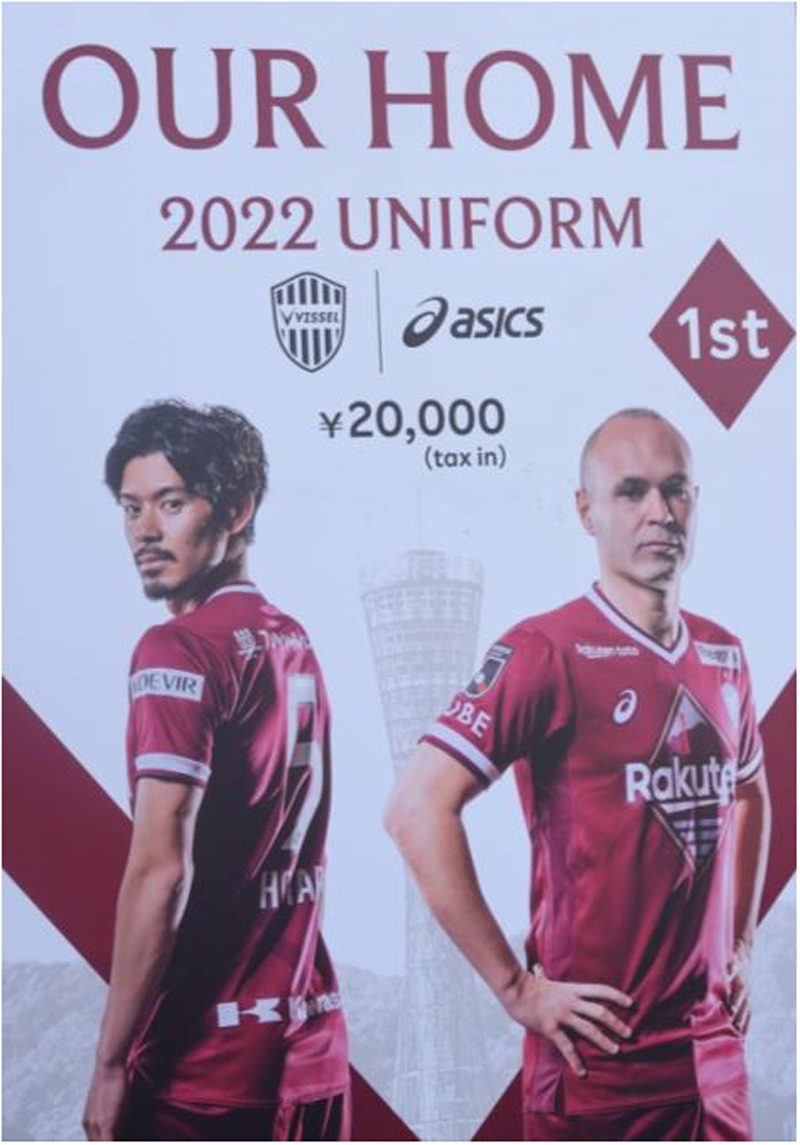
A billboard outside the stadium advertising the 2022 season's uniform.

Focusing here on structural support, the fan seems to prefer a minimal use of the body, prioritizing the display of clothing and merchandise, the act of watching the match and specific players, and only occasional moments of physical interaction with nearby spectators. Communication with strangers is minimal; indeed, in moments when the *gōru ura* supporters are not singing, one may experience uncanny silences in the stands. Within this context, the possession of totemic^6^ items to be displayed during the match becomes the crucial element in manifesting an emotional bond with the supported club. Objects themselves are subject to a hierarchy. For example, an official jersey of a particular player, signed by that player, demonstrates stronger emotional attachment, insofar as it shows the fan has invested extra time and money to attend training sessions or special events organized by the club (Figure 2). Another case is wearing an original vintage jersey of the club, an act that signifies the historical depth of one's attachment. Mari, a female supporter in her forties and a self-proclaimed hardcore fan, admitted quite proudly that her main way to support Vissel Kobe is through financial support.

“[At the stadium], it is of utmost importance to spend money. Vissel Kobe is like a religion; I must buy the jerseys, spend money. When I travel, I use Rakuten Travel, and for online purchases, I use Rakuten Ichiba. No true fan would use Amazon.” (Mari, August 23rd 2023)

In this case, the range of consumption is not limited to the club itself, but also to all the main sponsors. In the case of Vissel Kobe, the club and the main sponsors share the same person at the top, the businessman Mikitani Hiroshi. Therefore, any consumption related to the Rakuten Group is here seen as way of supporting the club as well. An emblematic aspect of Mari's way of supporting is one of her biggest club-related aspirations:

“I dream that one day my clinic becomes an official sponsor of Vissel Kobe. I would love to see my billboard on the field.” (Mari, August 23rd 2023)

As the owner of the clinic where she also works as a medical professional, she dreams of seeing her business displayed in the stadium as one of the club's sponsors. Having her contribution to the club officially materialized as a sponsor's billboard at the edge of the football ground would represent the apex of her commitment as a hardcore fan.

This case helps to illustrate how, within structural forms of support, a strong individualistic component can be observed in the dynamics of fandom. Supporters tend to focus heavily on purchasing goods and financially supporting clubs in various ways. With less emphasis put on physical and body-centered supporting behavior, the totems^6^ adorning their body (jerseys, mufflers, pins or gadgets), become the main way of materializing the attachment toward the club. The event itself nevertheless represents an escape from the structured routines of everyday life and provides a setting in which the *free-time-self* can be nurtured through the possession of objects imbued with strong symbolic value and their public display within the stadium. Undoubtedly, the sense of being part of a collective dynamic—though preferring a “passive” approach rather than a bodily-active participation—and the sharing of leisure time with members of one's family unit play an important role for those who do not attend the stadium alone. Yet even in these cases, the consumerist component appears to remain central to the perception of well-being within an arena where, semiotically, everything feels tied to consumption.

## 8 Anti-structural well-being: *gōru ura's* hardcore fans

As mentioned previously, the area behind the goals, known in Japanese as *gōru ura* (or *gōru no ura*), is associated with organized support, the most hardcore fans, and the most physical and spectacular forms of fandom, such as choreographed body movements or staged visual displays. Each club has several organized supporter groups of varying size, generally coordinated by a main group responsible for managing and directing the cheering. In the case of Vissel Kobe, the principal group is called *11Stones*, consisting of around forty active members. Belonging to such groups entails a significant commitment to attending as many matches as possible, both home and away. Beyond participation in games, these supporters also dedicate their free time to preparing choreographies, banners, and flags during weekdays or on weekends during the off-season period^7^. This type of support can be considered *anti-structural* for several reasons: a) their activity within the spatial-temporal frame of the football match is heavily based on physical performances (jumping, clapping, chanting, etc.), reshaping social norms of interpersonal distance and displaying behaviors that structural supporters often perceive as socially bordering on the unacceptable; b) supporting, its organization, away travel, and all related activities require a considerable investment of time and physical energy, ending up filling leisure time almost entirely with football-related activities. In becoming almost a second job, the conventional logic of time divided between work and leisure is replaced by a new rhythm alternating between work and the football match.

For most people, work is the most important thing, but for me the hierarchy is: football, family, and work in last place. (11Stones, Veteran supporter)

Consequently, the anti-structural nature of their mode of experiencing the football match is also reflected in the ways in which well-being is attained through this *contemporary capitalistic ritual*. Unlike structural supporters, who often attend the stadium alone or with their family unit, these fans are characterized by their tendency to operate collectively, a dynamic that leads many members to form important friendship ties within the group itself. As a result, rather than deriving happiness directly from the cheering activities (which are often tiring and stressful to organize), supporters seem to draw well-being from the bonds of companionship, from the regularity of weekly support, from the accomplishment of choreographic preparations, and from the successful performances of the club.

Another difference concerns their relationship with the purchase of club-related material goods. As an organized group, the *11Stones* have their own personalized dress code, which often excludes official merchandise altogether: original-design shirts and *11Stones* scarves. This does not mean, however, that anti-structural supporters never buy official club products. They may do so—sometimes even annually—but such purchases usually carry a specific motivation. Reasons may include the presence of a particular player who most clearly embodies their football ideals, or the release of commemorative jerseys marking the victory of a trophy or the anniversary of the club's founding.

**Figure 3:**
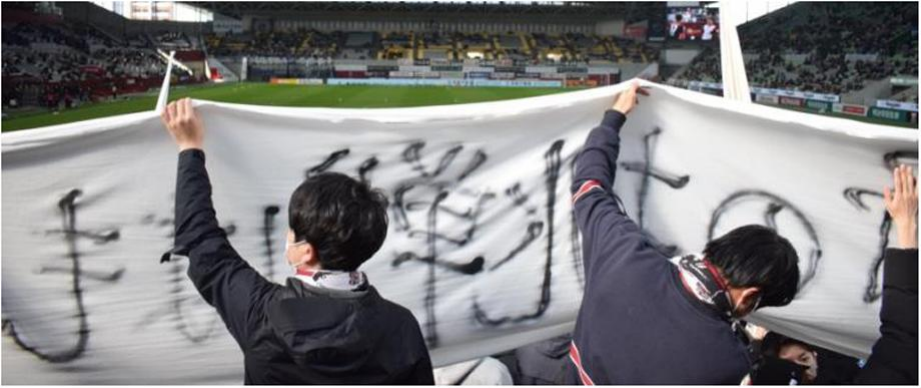
Supporters holding a banner during the team's warm-up session. (Photo by the author, February 26th, 2022).

For a *gōru ura* supporter, a regular league match begins at least five hours before kick-off. A meeting on the planned activities for the match marks the start of the day at the stadium, after which supporters faithfully follow the schedule to complete all necessary preparations. The required pre-match tasks depend on the specific game. For matches considered particularly important—such as derbies^8^ or finals—large-scale choreographies are organized, sometimes even requiring mobilization several days in advance. In other cases, for example when commemorating events related to specific players (appearance records, goal-scoring milestones, national team call-ups, etc.), the pre-match period is also used to produce personalized banners with written messages for the athletes (*messēji maku*) (Figure 3).

Preparations common to every match mainly concern the decoration of the entire *gōru ura* with horizontal banners (*danmaku*). Once all preparations are complete, supporters divide into small groups and share a meal while waiting for the gates to open. These small moments of pause and conviviality are fundamental to the imaginary of the anti-structural supporter, as they strengthen bonds with other group members and contribute to the establishment of hierarchies within the community. From this perspective, another highly symbolic moment for organized supporters is the away game^9^. When these involve long journeys, the group typically organizes itself to depart the night before in medium-sized vans. In addition to the many hours of travel, the away trip provides ample time for exchanging ideas about the organization of support, discussing the progress of the league, and creating shared memories (*omoide*) with fellow members. The away trip also functions as a kind of initiation ritual, serving as proof of one's seriousness of intent as a supporter: if one is willing to travel overnight and sacrifice sleep for the sake of supporting the team, one is recognized as a hardcore fan.

Whether at home or away, the central phase of *gōru ura* supporters’ activities coincides with the main event of the day, the football match itself. In this phase, their efforts focus on unconditional support for the players, beginning with their entrance onto the pitch, continuing through the warm-up, and reaching a climax during the ninety minutes of play. The support of organized fans is distinguished by an intensity unmatched in any other section of the stadium. The reason lies in their philosophy of support: these fans view European and South American football cultures as the models to be emulated, and thus their cheering activities seek to replicate, in both passion and intensity, these imagined foreign realities. Consequently, support is expressed symbolically through a masculine use of the body, involving vigorous movements, vocal force, and physical intensity. Ideally, their aspiration is to spread this philosophy throughout the entire *gōru ura*, a difficult task given the strong presence of structural supporters even within this area dedicated to cheering.

**Figure 4:**
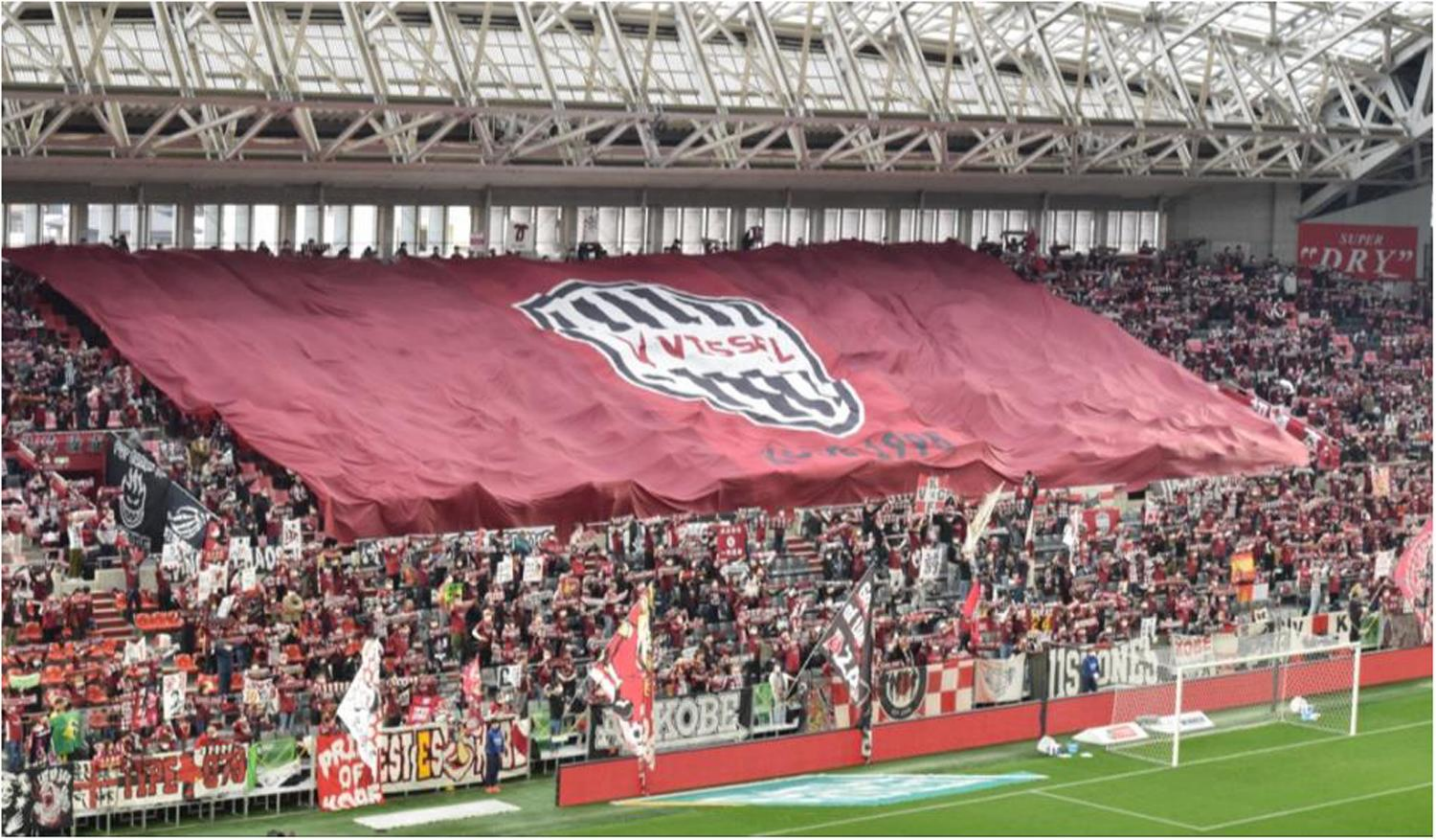
A large-scale flag choreography organized by the gōru ura supporters during the pre-match moments. (Photo by author, February 18th, 2023).

In fact, protests from so-called “regular” supporters^10^ are common regarding the extensive use of flags in the center of the *gōru ura*. The main reason is the impossibility of watching the match, as their view is obstructed either by the flags of the organized supporters or by the arms raised for choreographies during the game (Figure 4). Such protests highlight the characteristics of *structural support*, which in these cases gives priority to the individual's experience at the expense of the collective ritual of the *gōru ura*. Other “incidents” of this type occur when matches do not have assigned seating, and non-organized supporters attempt to position themselves in the center of the *gōru ura* or in the front rows (usually occupied by organized groups) in order to see their favorite players up close, or when they refuse to participate in group choreographies, preferring instead to take photographs of the players or to display their own personal banner (*gēto furaggu*) dedicated to a specific athlete.

### 8.1 Negotiating anti-structure within the neoliberal cultural frame

Given that all social actors enact their ritual patterns—whether structural or anti-structural—within the symbolic and normative boundaries shaped by contemporary capitalist ritual dynamics, it is legitimate to question the extent to which anti-structural modalities can truly be considered resistant to the neoliberal status quo. After all, hardcore supporters are also embedded in consumerist patterns, investing annually in goods and merchandise much like those they label as “regular” supporters.

However, to fully grasp the nature of their *anti-structurality* and their resistance to dominant values, it is essential to recall a central feature of ritual itself: liminality.^11^ Ritual space and time provide social actors with the opportunity to symbolically negotiate identities, hierarchies, and social roles, allowing them to momentarily detach from the passivity and subjugation imposed by the capitalist cycle and to adopt a more active, agentive stance. While the structural supporter accepts the grammar of the work-time/leisure-time divide and experiences the football match as a “ritualized consumer”—for whom financial transactions acquire symbolic meaning—the anti-structural supporter expresses dissatisfaction with what is perceived as a passive and emotionally detached mode of participation. To summarize, several key elements are at play in challenging the structural values of the neoliberal order:

1. **Free time as commitment rather than relaxation:** free time is not conceived as a space for rest, but as a site of *serious* commitment. Because work is not perceived as an existentially meaningful activity, an inversion of hierarchies occurs: the football match—and leisure time more broadly—acquires greater importance than weekday working activities. The match becomes a “serious” practice, oriented toward actively supporting the players through chants and choreographies. This commitment requires a substantial investment of time and money, yet yields only symbolic returns, such as a sense of agency during the match, happiness in the case of a positive outcome, and a strong feeling of belonging to a community.
2. **From individualism to community orientation:** *gōru ura* supporters understand cheering as the outcome of collective effort. Actions undertaken solely for individual gratification are therefore despised, while individual initiatives that produce positive effects for the group are encouraged and valued. This stance marks a clear rupture with the practices of most regular supporters, who statistically tend to attend matches alone or in small family units, and often seek individualistic advantages. In this sense, mainstream stadium attendance mirrors the individualism and social isolation that often characterize neoliberal societies, whereas *gōru ura* cheering foregrounds collective coordination and mutual dependence.
3. **Ritualized emotional investment**: where structural cheering tends to emphasize the symbolic dimension of financial transactions and merchandise consumption (economic investment)—often accompanied by limited emotional display and minimal verbal or physical interaction—anti-structural cheering involves a highly ritualized expression of emotions (emotional investment). Emotional display varies according to the specific match and the team's performance, both within the game and across the season. Broadly speaking, major victories are marked by euphoric joy, conveyed through intense physical interaction such as collective embraces and handshakes. Conversely, defeats (especially in important matches) are often followed by expressions of sadness or anger, frequently taking the form of prolonged collective silence, during which no one dares to break the shared sense of despair for several minutes.

It is important to note that the *anti-structurality* of *gōru ura* is not negotiated solely in relation to neoliberalism. Social structure—in this case, Japanese society—should be understood as the broader system of norms, moralities, and expectations upon which institutions, communities, and individuals structurally rely. From this perspective, *anti-structurality* can be interpreted as a ritual mode of behavior that expresses resistance not only to neoliberal logics, but also to a wider set of societal norms deeply embedded in Japanese social life. These include conventions regulating interpersonal distance, social hierarchies, social behavior, bodily exposure, and emotional restraint. As argued earlier in this dissertation, such norms have been increasingly shaped and reinforced by the pervasive influence of neoliberal culture. Seen in this light, *gōru ura* practices do not constitute an external alternative to neoliberal capitalism, but rather a ritualized mode of negotiation with both neoliberal and culturally specific social structures. Their *anti-structurality* lies not in escaping these frameworks, but in temporarily suspending, reworking, and contesting them through embodied, collective, and emotionally intensive practices. This ambivalent positioning is precisely what makes such rituals analytically productive for understanding contemporary configurations of well-being.

## 9 Conclusions

This paper has examined how neoliberal capitalism shapes the symbolic and experiential dimensions of well-being through contemporary ritual practice. By conceptualizing capitalism as a belief system rather than a mere economic order, the paper argues that rituals embedded in capitalist logics constitute a central site where values, identities, and experiences of happiness are produced and negotiated. This perspective reframes well-being not as an autonomous state but as a culturally mediated construct deeply entangled with neoliberal temporalities and moral frameworks.

The ethnographic case of Japanese football provides a paradigmatic example. Since its inception, the J.League was planned as a neoliberal project: a cultural-financial product sustained by marketing, consumerism, and global symbolic capital. Within this framework, supporters perform a variety of ritual practices that reveal how capitalism organizes both leisure and identity. Structural supporters embody the individualistic ethos of neoliberalism, situating well-being in the possession and display of consumer goods and in the privatized enjoyment of leisure. Conversely, the *gōru ura*'s organized supporters articulate an anti-structural mode of participation, where collective performance, solidarity, and intense corporeal engagement become primary sources of well-being. Yet both modalities remain tethered to the capitalist grammar of free time, underscoring the pervasiveness of neoliberal values even in spaces that appear resistant.

These findings advance three key claims. First, they highlight the inadequacy of treating rituals in contemporary societies as vestiges of tradition or as reducible to consumerist practices. Rituals remain central symbolic mechanisms that adapt to shifting socio-economic orders, structuring both individual and collective horizons of meaning. Second, they demonstrate that the pursuit of well-being in capitalist societies cannot be detached from the symbolic grammars of neoliberalism. Whether through consumption or collective effervescence, happiness is pursued within frameworks that reproduce the capitalist temporal cycle of work and leisure. Third, they reveal how mega-events like professional football function not only as entertainment but also as infrastructures of value production, embedding capitalist logics into the very affective registers of collective life.

The broader implication is that well-being in neoliberal societies is never external to capitalism but is constituted through it. Football mega-events exemplify how capitalism operates affectively and symbolically, sustaining its hegemony by providing both moments of relief and opportunities for belonging. At the same time, the presence of anti-structural practices points to the potential of rituals to generate counter-hegemonic spaces, though always in negotiation with the wider capitalist order. This dialectic of reproduction and resistance suggests that rituals remain critical sites for understanding how people navigate the contradictions of neoliberal modernity.

By proposing *contemporary capitalistic rituals* as an analytical category, this paper contributes to debates on the socio-cultural infrastructures of well-being and the reconfiguration of public culture in the global era. Extending this framework beyond sport (digital communities, environmental movements, or urban festivals) could offer potential new avenues for future research. In doing so, anthropology can illuminate not only how rituals reproduce neoliberal capitalism but also how they open spaces for reimagining collective life and alternative visions of well-being.

## Data Availability

The raw data supporting the conclusions of this article are not publicly available due to privacy concerns. Requests to access the data should be directed to the author.

## Footnotes

^1^In this article, “football” refers to what is known in American English as “soccer.”

^2^An abbreviation of the expression *bukatsudō* (lit. “extracurricular activities”), *bukatsu* refers to after-school activities or corporative sports clubs that allow students to engage in music, art, culture, and sport. Today, some schools and universities are particularly renowned for the high quality of their sports *bukatsu*. Despite the rise of youth academies managed by professional clubs, for many decades *bukatsu* functioned as a professionalizing institution, enabling numerous players to reach elite levels both nationally and internationally.

^3^ https://aboutj.jleague.jp/corporate/en/about_j/profile_j/

^4^According to surveys conducted by the J.League (52) and Deloitte Tohmatsu Group (51), between 54.3% and 62.5% of supporters attend matches with a family member, while between 17.3% and 26% go to the stadium alone.

^5^Official fan clubs are paid memberships recognized by the clubs, offering benefits such as priority ticket sales, access to events, occasional discounts, and different tiers with varying privileges.

^6^In cultural anthropology (see Totemism), a totem is a natural entity or object of strong symbolic value (identity, ancestry, and group cohesion). In this context, the term refers to objects associated with the club that symbolize the supporter's emotional attachment to the team.

^7^The off-season period refers to the months when no matches are scheduled, during which supporters can organize meetings and workshop sessions in preparation for the following season.

^8^In football jargon, a derby refers to a match between two clubs representing geographical areas with a long-standing history of rivalry. Derbies are often contested between clubs from the same city but different neighborhoods, or between clubs from the same region or adjacent localities.

^9^Away games are matches played at the opponent's stadium. Depending on the location of the host club, the trip for the supporters can take up to eight hours by car. Since Japan is an archipelago, travel by boat or plane is sometimes required.

^10^Organized supporters usually refer to the rest of the fans or spectators as *ippantekina sapōtā* (lit. “regular supporters”).

^11^Liminality is a key concept in ritual theory proposed by the British anthropologist Victor Turner in his work *The Ritual Process: Structure and Anti-Structure (1969)*. In his analysis, rites of passage are typically structured into three phases: separation, liminality, and reaggregation. During the liminal phase, the entity subjected to the ritual (whether a person, a temporal moment, or a spatial setting) enters a ritual condition in which shared social norms and rules are temporarily suspended, inverted, or renegotiated.
